# Report of the Diseases of Edinburgh, for May 1808

**Published:** 1808-07

**Authors:** John Roberton

**Affiliations:** Surgeon


### *Report of the Diseases of Edinburgh, for May* 1808; *by* Mr. John Roberton, *Surgeon.*

During the first four days of this month, we had frequent showers of rain, succeeded by cold easterly winds, which, as usual in this city, when the wind blows with any degree of violence from that quarter, was attended with fogs, particularly towards the afternoon. About the end of the first week, the thunder was for two days almost constant, immediately succeeded by excessively heavy rains and hail, and the cold easterly winds which had subsided, seemed again to prevail, which rendered the weather very unpleasant. It was supposed by those whose business it is more particularly to attend to these matters, that the uncommon falls of snow which lately took place in this part of the world, at such an advanced period of the season, would prove very injurious to vegetation, and consequently render our crops not only later than usual, but that they would completely destroy a great porportion of them. The wonderfully rapid progress, however, which vegetation made, immediately after the general thaw, which took place last month, yielded an additional proof, that early vegetation with us is uniformly attended by greater chances of suffering from our frequent cold, and often rapid changes from hot to cold days, during the months of March, April, and even May, than when, as in the present season, the uniform gloom of winter has been protracted to an unusually late period. It was likewise supposed that the thunder, hail, and rain, about the beginning of the present month, would contribute very much to the destruction of the tender fruit, &c.; but, independent of all these seemingly unfavorable occurences, our fields, have not, for many years past, displayed a more beautiful appearance, or yielded such a prospect of luxuriant crops as they now, all over this part of the country, exhibit. Near the middle of the month the weather became enchantingly fine, and for several days the thermometer stood high. About the last third of the month, however, cold easterly winds again annoyed us; and the barometer, which, although low about the beginning of the month, had risen considerably during the few good days we experienced, now fell very fast, and heavy rains again began and continued for two or three days. After this, however, till the termination of the month, the weather, during the day, continued tolerably warm, but the nights were cold and disagreeable.

Athough the Ophthalmia, mentioned in my last Report, was, indeed, very general while it lasted, and, in some instances, very severe, it rapidly abated in its violence about the beginning of the present month; and before the end of the first third of it, scarcely any cases of this disease occured in practice.

I took notice of a distinction between the violence of its effects in scrofulous patients, and those in whom no such state of the system could be traced. With some patients of the former description, I have met with cases of Ophthalmia, not so violent in their nature, as extremely tedious in their their removal. After the inflammatory symptoms seemed nearly abated, the eyes often remained long unable to perform their healthy functions. Distinct red blotches appeared on various parts of the balls of the eyes, as if occasioned by the rupture of small blood vessels; and a constant and very plentiful discharge of a thin watery fluid, often so acrid in its nature as to irritate the neighbouring parts, was discharged from the eyes, which proved very troublesome. In such patients I have not experienced much benefit from a continuance of the same treatment which I recommended in my last Report, and which I advised during the active state of the inflammation in such complaints. The repeated application of blisters, as near the affected eye as possible, has, among my patients, proved of greater service, with the addition of the daily use of cold sea-bathing. This last advantage, viz. sea bathing, opportunities of which, from our local situation we possess in so eminent a degree, is certainly of the greatest utility, as well to the healthy as to the convalescent. We can, in about two hours, in a very gentle walk, from almost any part of Edinburgh, reach the sea, bathe, and return sensibly invigorated by it. For those whose complaints, or general state of constitution, require warm sea-bathing, we are most conveniently accommodated at Porto Bello, on the sea shore, about three miles from any part of the city. These, with the pleasant lodgings and delightful scenery on every hand, render such a situation invaluable. One disadvantage, however, which I hope will soon be remedied by the proprietors of these bathing places, is the complete want of proper accommodation for those who wish to enjoy the benefit of bathing without being exposed to every person. Bathing machines are, indeed, provided; but, while employed, they are are so closely huddled together that the bathers seem to belong to one party. Our southern neighbours are much better accommodated than we are with these machines, in the comparatively dirty bathing in some parts of the Thames. By carrying the same principle adopted by them to a greater extent, viz. that of attaching a canvas covering to one end of the machine, we might be still more effectually prevented from making a public exhibition of ourselves than they are. In short, whilst bathing, by this very easy plan, a whole family, or any individual of it, may command any extent of room, while they completely prevent what I here complain of; and the expence of such an equipage would be so extremely trifling, as to preclude the necessity of excuse in in that way. I hope, if this custom be not generally adopted, those gentlemen, with families, who frequent our bathing places, will, upon this principle, or any other that may seem to them better calculated to answer the end I have proposed, set an example to others, which will prevent many, particularly females, from urging those scruples against sea bathing, which I know are very common in this, as, I believe, in every other place similarly circumstanced. Were such measures adopted here, and the plan made generally public, I have no hesitation in saying, that the neighbourhood of Edinburgh would become one of the most fashionable bathing places in the island. The lodging and living of every description are, comparatively speaking, cheap, excellent, and in the greatest variety; and the fresh sea breeze, with the rural walks, may, in the course of a few minutes, be changed for those gay circles of beauty and fashion which the city of Edinburgh can so justly boast of. Thus every description of individuals, of whatever way of thinking, may frequently, in the course of the same day, be alternately gratified with every different sort of pleasure of which the human mind is susceptible.

Almost immediately after the excessively heavy falls of rain, which occurred near the beginning of the month, inflammatory complaints, particularly Pneumonia and Enteritis seemed to recur in many instances with considerable violence.

Blood-letting, with the administration of saline cathartics, in both these affections, was had recourse to with very good effect. In some cases, however, where the patient was of a weakly habit of body, I have preferred the application of a large blister to the abdomen or chest, as these parts happened to be individually affected. From this caution I have derived the greatest success, which to me is the most satisfactory proof of the necessity of greater attention being paid than is at all times done in practice, to many little particulars, not only connected with the existing disease, but with the habit of body, manner of living, &c. of the patient for a considerable length of time, previous to his being affected with such complaints. The physician who prescribes, without attending to these cautions, is a mere mechanic, and totally unworthy of public confidence in the line of his profession. The blister, under the above circumstances, a few hours, or even a day after it has been applied, accelerates the pulse, and renders it fuller; but this soon abates, and relief of the symptoms is is almost always obtained. I have, about this time, particularly observed, that a great proportion of those children, previously very much debilitated by the measles, were peculiarly liable to inflammation of the bowels. In such cases, blistering alone was found serviceable. I have heard of some cases in which bleeding was employed, the effect of which was want of that success, which it is the wish of every medical man should attend his prescriptions. One complaint, as in the present instance, seems to predispose the system to the attack of another, sometimes nearly similar to the formet, sometimes of an apparently different description. Parts in a state of morbid action, or in any way debilitated by any previous disease, seem, during certain epidemics, more particularly liable to be affected in a very severe degree, than almost in any instance where such epidemics attack parts apparently sound in other respects.

These insidious diseases may sometimes lurk in the system without being detected, as happened in the following case: the patient, aged 40 years, was remarkably stout, and apparently enjoyed a state of good health, though for some years past he has been subject to frequent attacks of colic, and also to diarrhœa. His last illness was only of six days continuance. The symptoms were those usual in inflammation of the bowels, only that the costiveness was more obstinate. On opening the body, the illeum, where it terminates in the colon, for about six inches in length, was found surrounded with a scirrhous tumour, which besides completely obstructing the passage, was greatly inflamed, and around its edges gangrenous, being covered with a blackish coloured matter, and having lost its tenacity so as to be easily penetrated by the pressure of the finger. The inflammation had extended to those parts of the intestines which were contiguous to the seat of the disease. One of the mesenteric glands was swelled to the size of a pigeon's egg; none of the other viscera were at all diseased. As, from the symptoms of the above patient, during his last illness, no such affection as that of which he died could be suspected, it furnishes us with an additional proof of the extreme caution which at all times ought to be observed in forming our prognosis of *any disease.*

No. 4, *St. James's Street.*

